# Molecular Imaging of Nonsmall Cell Lung Carcinomas Expressing Active Mutant EGFR Kinase Using PET with [^124^I]-Morpholino-IPQA

**DOI:** 10.1155/2013/549359

**Published:** 2013-07-17

**Authors:** Skye Hsin-Hsien Yeh, Chien-Feng Lin, Fan-Lin Kong, Hsin-Ell Wang, Ya-Ju Hsieh, Juri G. Gelovani, Ren-Shyan Liu

**Affiliations:** ^1^National PET/Cyclotron Center, Department of Nuclear Medicine, Taipei Veterans General Hospital, Taiwan; ^2^Department of Biomedical Imaging and Radiological Sciences, National Yang-Ming University, Taipei, Taiwan; ^3^Biophotonic and Molecular Imaging Research Center, National Yang-Ming University, Taipei, Taiwan; ^4^Taiwan Mouse Clinic, National Comprehensive Mouse Phenotyping and Drug Testing Center, Taiwan; ^5^Department of Experimental Diagnostic Imaging, The University of Texas MD Anderson Cancer Center, TX, USA; ^6^Department of Medicinal Imaging and Radiological Sciences, College of Health Sciences, Kaohsiung Medical University, Kaohsiung, Taiwan; ^7^Department of Biomedical Engineering, Wayne State University, MI, USA; ^8^Department of Nuclear Medicine, National Yang-Ming University School of Medicine, Taiwan

## Abstract

Mutations in the kinase domain of epidermal growth factor receptor (EGFR) have high levels of basal receptor phosphorylation and are associated with clinical responsiveness to Iressa in patients with nonsmall cell lung cancer (NSCLC). This study aimed to assess the feasibility of morpholino-[^124^I]IPQA derivative as an *in vivo* PET imaging tool for the expression of different EGFR mutants in NSCLC. *In vitro* radiotracer accumulation and washout studies demonstrated a rapid accumulation and progressive retention after washout of morpholino-[^131^I]IPQA derivative in high EGFR-expressing H1299 NSCLC derivative cell lines (L858R and E746-A750 del cell lines), but not in EGFR-transfected H1299 cell line and vector-transfected H1299 cell line. Using the morpholino-[^124^I]IPQA derivative, we obtained noninvasive microPET images of EGFR activity in L858R and E746-A750 del subcutaneous tumor xenografts, but not in subcutaneous tumor xenografts grown form control cell line. Different EGFR mutant (activity) tumors have a different morpholino-[^**∗**^I]IPQA derivative uptake. However, it still needs to modify the structure of IPQA to increase its water solubility and reduce hepatobiliary clearance. Morpholino-[^124^I]IPQA derivative may be a potential probe for selection of the candidate patients suffering from NSCLC for the small molecule tyrosine kinase inhibitor therapy (e.g., Iressa) in the future.

## 1. Introduction

Nonsmall cell lung cancer (NSCLC) represents the majority of lung cancers [1]. Lung cancer treatment depends on several factors including tumor type, size, and patient's health. Surgery, radiation therapy, and chemotherapy using tumor specific targeted agents such as vascular endothelial growth factor (VEGF) inhibitor bevacizumab and epidermal growth factor receptor-tyrosine kinase inhibitors (EGFR TKIs) are the primary tools for treating lung cancer. These targeted agents are initially effective in certain small subpopulations of patients, but eventually nearly all patients turn out to be resistant to the further treatments [2]. The limitations in efficacy and safety associated with the existing treatments for NSCLC underscore the need for novel biomarkers and imaging approaches for identification of patients who may benefit from particular therapeutic agents and approaches with improved efficacy and safety profiles.

The importance of EGFR signaling pathway in the development and progression of NSCLC has been widely recognized [3, 4]. EGFR overexpression is observed in tumors of more than 60% of patients with metastatic NSCLC and correlates with poor prognosis [5]. These observations have provided a rationale for the development of novel anticancer agents that target EGFR. Two classes of EGFR-targeted agents are currently being tested in clinical trials: antibodies that bind to the extracellular domain of EGFR (i.e., cetuximab, panitumumab, matuzumab, nimotuzumab, etc.) and small molecular inhibitors that bind to the ATP-binding site of the EGFR tyrosine kinase (i.e., gefitinib, erlotinib, lapatinib, canertinib, etc.) [6]. Clinical trials have revealed significant variability in response to EGFR TKI gefitinib (Iressa), with approximately 10% of Caucasian patients, 25–30% of Japanese patients, and 57% of Taiwanese patients [5, 7–13]. Sequencing test of the *EGFR* gene is associated with a majority of tumors responding to EGFR kinase inhibitors harbor mutations in the kinase domain of EGFR [9, 14, 15]. The response rate to gefitinib and erlotinib in patients with tumors exhibiting activating mutations of EGFR is approximately 75%, suggesting that these mutations, at least in part, may have caused malignant transformation and contribute in large to the tumor maintenance pathway [16, 17]. Two subtypes of activating EGFR mutations have been described: tyrosine kinase domain mutation (45%–50%) in EGFR exons 18–24 and truncating mutations involving exon 2 to 7. The most frequently detected alterations are small deletions in exon 19 (35%–45%) that eliminate amino acids 747–750 (Leu-Arg-Glu-Ala), located around the active site of the tyrosine kinase, and point mutations in exon 21 that result in the amino acid substitution Leu858→Arg, a residue located in the activation loop [9]. Mutations are most frequently detected in a subpopulation of NSCLC patients with characteristics associated with a better treatment outcome: women, nonsmokers, patients of southeast Asian and Japanese origins, and patients with adenocarcinoma histology and, in particular, bronchioloalveolar carcinoma [18, 19]. The mutational status of EFGR kinase could be considered as a positive predictive biomarker of response to NSCLC [20, 21].

Considerable efforts have been made over the past decade to develop radiolabeled agents for noninvasive imaging of EGFR expression and activity. These agents include radiolabeled antibodies to EGFR and radiolabeled small molecular compounds based on structures of known EGFR TKIs [22]. Most of the radiolabeled agents for molecular imaging with positron emission tomography (PET) are derived from 4-(anilino)quinazoline pharmacophore [23, 24], which includes ML series [21, 22, 25–27], as well as [4-(3-[^124^I]iodoani-lino)-quinazolin-6-yl]-amide-(3-morpholin-4-yl-propyl)-amide ([^124^I]IPQA) [28], [^18^F]gefinitib [29], [^11^C]PD153035 [30], and [^11^C]erlotinib [31]. Recently, clinical studies with [^11^C]PD153035 have demonstrated some promise for imaging EGFR expression in NSCLC patients [32]; however, none of these imaging agents exhibits selectivity for detection of NSCLC expressing active mutant EGFR kinases or EGFR kinase mutations that confer resistance to inhibitors that are currently used in clinical practice. Therefore, we have been developing a PET radiotracer with preferential binding to active mutant EGFR kinases, not to EGFR kinase mutants conferring resistance to current small molecular inhibitors (i.e., gefitinib). PET imaging using such a selective radiolabeled agent should allow for visualization of primary and metastatic tumor lesions driven by activating mutations in EGFR kinase and selection of patients who may benefit from therapy with EGFR kinase inhibitors. Also, after the initial course of treatment with EGFR inhibitors, repetitive imaging with such a selective radiotracer could be used for monitoring the development of tumor lesions with acquired mutations (i.e., T790 M) conferring resistance to EGFR inhibitors.

Here, we describe the [4-(3-[^124^I]iodoani-lino)-quinazolin-6-yl]-amide-(3-morpholin-4-yl-propyl)-amide ([^124^I]morpholino-IPQA) as a PET imaging agent with increased selectivity and irreversible binding to active mutant L858R and E746-A750 del EGFR kinase, which allows for noninvasive detection of NSCLC in mouse xenografts harboring this mutation.

## 2. Materials and Methods

### 2.1. Radiosynthesis of Morpholino-[^124^I]IPQA Derivative

Morpholino-[^124^I]IPQA derivative was synthesized from but-2-enedioic acid(3-morpholin-4-propyl)-amide[4-(3-tributylstannyl-phenyl-amino)-quinazolin-6-yl]-amide (morpholino-IPQA derivative), as described previously [28]. The radiosynthesis of morpholino-[^124^I]IPQA derivative was modified according to the earlier study [33]. The tin precursor (100 *μ*g; from MD Anderson Cancer Center, Houston, TX, USA) was dissolved in 0.02 mL of methanol, and then the [^124^I]NaI (37~55 MBq) was added to the solution of precursor and vortexed followed by the addition of a 0.018 mL mixture of 30% hydrogen peroxide/acetate acid (1 : 3). The reaction mixture was vortexed for 1 min and then allowed to stand for 5 min. Saturated sodium bicarbonate (0.2 mL) and 2N sodium thiosulphate (0.12 mL) were added to quench the reaction, and the reaction mixture was then loaded to a plus C-18 Sep-Pak cartridge system. The C-18 cartridge system (preconditioned with 10 mL of ethanol, then 10 mL of water) was eluted with water (30 mL), followed by 20% ethanol/water (25 mL), 40% ethanol/water (25 mL), and 60% ethanol/water (25 mL). The majority of the product was isolated during the elution with 60% ethanol/water. Each fraction was collected and assayed for radioactivity measurement, and the fractions that contain the final product were assayed by radio-thin-layer chromatography (radio-TLC). The radiochemical purity was determined by radio-TLC (silica gel 60 F254; eluent: chloroform/methanol = 6/1). The collected products with sufficient purity were combined and evaporated.

### 2.2. Radiosynthesis of Morpholino-[^131^I]IPQA Derivative

No carrier-added morpholino-[^131^I]IPQA derivative was prepared using the same procedure as that of morpholino-[^124^I]IPQA (50 *μ*g of the tin precursor was used). The radiolabeled product was isolated in 65% radiochemical yield (decay corrected) with a radiochemical purity of 92.3%. 

### 2.3. Tumor Cell Lines

Human NSCLC cell line H1299 with 4 different levels of wild-type (WT) or mutant EGFR expression was selected: (a) L858R EGFR (point mutation in exon 21); (b) E746-A750 del EGFR (in frame deletion); (c) EGFR-transfected (wild-type); (d) vector-transfected (study control). 

Cells were grown in flasks with RPMI 1640 with 10% FBS and antibiotics at 37°C in humidified atmosphere with 5% CO_2_. Cells were kept in the log phase of proliferative activity. The L858R, E746-A750del, EGFR-transfected, and vector-transfected H1299 cells were kind gifts from Drs. Shih-Feng Tsai and Yi-Rong Chen (The National Health Research Institutes, Miaoli, Taiwan).

### 2.4. Irreversible Binding of Morpholino-[^124^I]IPQA to Active Mutant EGFR Kinase Domain

The irreversible and covalent bindings of morpholino-[^131^I]IPQA to the EGFR kinase domain were evaluated in H1299 derivatives cells. The cells were grown in a 15 cm culture dish until 60–70% confluency and then incubated for one hour in fresh culture medium supplemented with 20% FBS and morpholino-[^131^I]IPQA at 0.37 MBq/mL. Thereafter, the cells were harvested by scraping, pelleted by centrifugation at 1,000 rpm for 5 minutes, and lysed in 0.5 mL of buffer containing protein extraction reagent (Cytobuster, Novagen, USA) and aprotinin, leuppetin, pepstain (1 *μ*g/mL for each), Na_3_VO_4_, NaF, and PMSF (1 mM for each; Sigma-Aldrich, CA, USA). The cell lysate was cleared by 14,000 ×g centrifugation at 4°C for 15 minutes. The cell lysate supernatant was denatured by boiling with 4x Laemmli sample buffer and separated by sodium dodecyl sulfate polyacrylamide gel electrophoresis using precast 8% Tris HCl gel cassettes (BioRad, CA, USA). After transferring proteins into a minitank electroblotter device, the membrane was exposed to AX film (Konica, Japan) for seven days at room temperature to produce an autoradiogram of ^131^I-labeled protein bands. Thereafter, the nitrocellulose transfer membrane was immunostained with a rabbit polyclonal EGFR (1005)-sc-03 antibody (Santa Cruz, CA, USA) and visualized using the ECL kit (Amersham Biosciences, UK). The colocalization of ^131^I-labeled proteins in the autoradiogram with protein bands stained with anti-EGFR antibody was assessed.

### 2.5. *In Vitro *Radiotracer Uptake and Washout Assay

Radiotracer uptake and washout studies were performed in monolayer cultures of four NSCLC cell lines as described previously [28]. Briefly, tumor cells were grown in 15 cm culture dishes until 60~70% confluent, at which point the cells were exposed to the culture medium without FCS to induce serum starvation (to inhibit EGFR kinase activity). The radiotracer morpholino-[^131^I]IPQA at 0.18 MBq/mL was then added to fresh cell culture medium without FCS (serum-starved), and tumor cell monolayers were exposed to the radioactivity-containing medium for 5, 10, 20, 30, and 60 minutes. In the first part of the experiment, the cells were harvested by gentle scraping at different time intervals, pelleted by centrifugation (3,500 rpm for 2 minutes). The cell pellet and 0.1 mL of radioactive supernatant were weighed and assessed for radioactivity using a Packard 5500 gamma counter (Perkin-Elmer, CA, USA); the radioactivity concentration was expressed as cpm/g cells and cpm/mL medium, respectively. In the second part of the experiment, tumor cells exposed to the morpholino-[^131^I]IPQA containing medium for a given time interval (5, 10, 20, and 60 minutes) were washed with fresh (nonradioactive) medium for different time intervals before subsequent harvesting for measurement of retained radioactivity, as described above. Cells-to-medium radioactivity concentration ratios were calculated and plotted versus time to evaluate the radiotracer accumulation and washout kinetics.

### 2.6. Subcutaneous Tumor Xenografts and PET Image Acquisition and Data Analysis

NOD/SCID mice weighing about 25~30 g (*N* = 3) were injected subcutaneously into the left shoulder region with L858R, E746-A750 del, or wild-type EGFR-transfected H1299 cells (5 × 10^6^ cells/mouse). In the right shoulder region of each mouse, 5 × 10^6^ of vector-transfected H1299 cells were inoculated as a control tumor. The tumors grew to about 8~10 mm in diameter after 4 weeks. At this point, the mice were anesthetized (2% isoflurane/98% oxygen mixture) and injected intravenously with morpholino-[^124^I]IPQA (2.6 MBq/mouse). One-hour dynamic PET imaging was performed on MicroPET R4 system (Siemens, TN, USA) and followed with a 30-minute static imaging at 24 hours after i.v. administration of radiotracer. PET images were reconstructed using the ordered subsets expectation maximization iterative reconstruction algorithm. Regions of interest were drawn over tumors and other tissues of interest.

### 2.7. Biodistribution of Morpholino-[^131^I]IPQA Derivative in Mice

NOD/SCID mice (*n* = 3/time point) were injected subcutaneously into shoulders and limbs with L858R (8 × 10^6^ cells/mouse), E746-A750 del (8 × 10^6^ cells/mouse), wild-type EGFR-transfected (8 × 10^6^ cells/mouse), and vector-transfected H1299 cells (5 × 10^6^ cells/mouse). Three weeks later, each mouse was injected with 0.1 mL of a saline solution containing 2.6 MBq of morpholino-[^131^I]IPQA through the caudal vein. Animals were sacrificed by chloroform (Nacalai Tesque Inc., Japan) at different time points after i.v. administration of radiotracer. Organs of interest were collected and weighed, and the radioactivity was counted. The percent injected dose per gram of tissue (% ID/g) was calculated and recorded.

### 2.8. Statistical Analysis

Group data were expressed as average ± SE and compared using analysis of variance, regression analysis, and group and paired Student's *t* tests; a *P* value of <0.05 was considered statistically significant.

## 3. Results

### 3.1. Radiochemistry

**Figure 1:**
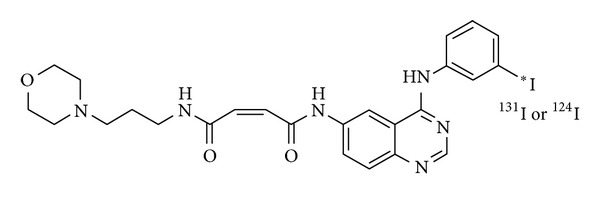
The chemical structure of the (E)-But2-enedioic acid [4-(3-[^124^I]iodoanilino)-quinazolin-6-yl]-amide-(3-morpholin-4-ylpropyl)-amide, termed as morpholino-[^124^I]IPQA.

The final product of morpholino-[^124^I]IPQA derivative (Figure 1) was dissolved in saline (with 10% ethanol added). Radio-TLC showed the Rf value of morpholino-[^131/124^I]IPQA derivative to be 0.5. The radiochemical yield was 50%, and the radiochemical purity was ≥90% (decay-corrected). Radio-high-performance liquid chromatography (radio-HPLC) using an Alltech Alltima C18LL column (250 × 4.6 mm; Fisher Scientific, USA) and a mobile phase consisting of 0.1 M acetate buffer (acetate/acetonitrile = 55/45) at a flow rate of 1.0 mL/min exhibited the product as a peak at about 10 minutes.

### 3.2. Irreversible and Specific Bindings of Morpholino-[^131^I]IPQA to the L858R and E746-A750 Del EGFR Mutations

**Figure 2:**
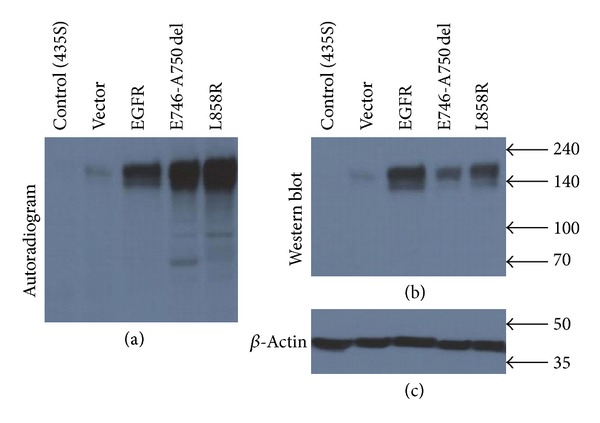
Assessment of irreversible binding of morpholino-[^131^I]IPQA to wild-type and mutant EGFR kinase in four types of human nonsmall cell lung carcinoma cell lines. (a) The autoradiography demonstrates the irreversible and covalent bindings of morpholino-[^131^I]IPQA to the EGFR kinase domain in different cell lines. (b) The same membrane was stained with anti-EGFR kinase antibody. A single band of radiolabeled protein corresponds to the predominate band of ~170 kDa.

The autoradiographic and western blot analysis was used to demonstrate the irreversible and covalent bindings of morpholino-[^131^I]IPQA in four types of NSCLC cells. The autographic electropherogram of protein extracts from these NSCLC cell lines that were pretreated with morpholino-[^131^I]IPQA demonstrated the preferential convent binding of morpholino-[^131^I]IPQA to the cells with L858R and E746-A750 del EGFR mutations (Figure 2(a)) These bands were correspondent with the 172 kDa protein band stained with anti-EGFR antibody (Figure 2(b)). The above observations collectively suggest that in EGFR-transfected cells (cells with wild-type EGFR overexpression) the lower radioactive intensity of morpholino-[^131^I]IPQA (Figure 2(a)) corresponds to a higher level of anti-EGFR immunoblotting (Figure 2(b)) compared to EGFR mutation cells.

The covalent binding of morpholino-[^131^I]IPQA to the H1299 vector-transfected and MB-435S (EFGR negative cells) was barely detected by autographic electropherogram (Figures 2(a) and 2(b)).

### 3.3. Preferential Accumulation of Morpholino-[^131^I]IPQA in NSCLC Cells with L858R and E746-A750 Del EGFR Mutations

**Figure 3:**
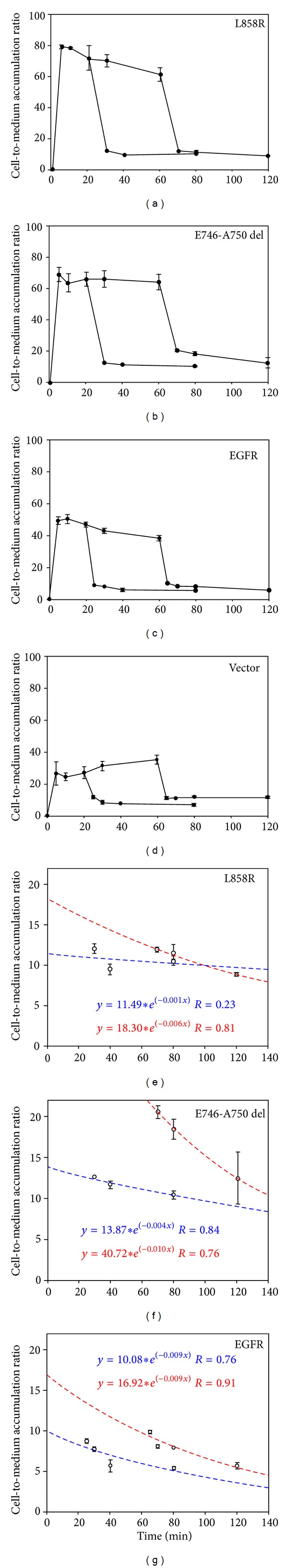
*In vitro* uptake and washout phases of morpholino-[^131^I]IPQA in L858R (a), E746-A750 del (b), wild-type EGFR-transfected (c), and EGFR-vector (d). Liner fits of radioactivity accumulation time points show the washout rate at short duration (20–80 minutes, blue dot line) and long duration (60–120 minutes, red dot line) after initial accumulation (from (e) to (g)). Coefficient *B* (slope) of the exponential washout = *A* × exp⁡^(−*BX*)^. Panels (a)–(d) are shown in full scale, and panels (e)–(g) are from the same data in panels (a)–(c) but are shown in smaller scale to visualize differences in washout results.

All four types of cells showed a rapid accumulation of morpholino-[^131^I]IPQA at the initial 10 minutes and thereafter reached a plateau at 60 minutes (Figures 3(a)–3(d)). In L858R and E746-A750 del cells, the cell-to-medium ratio (CMR) of morpholino-[^131^I]IPQA was about 60–65 at 60 minutes, which was ~1.6-fold higher than that of wild-type EGFR-transfected H1299 cells (Table 1).

In L858R and E746-A750 del cells, the loss of morpholino-[^131^I]IPQA accumulation could be characterized by a rapid washout, followed by a plateau, with a slow but slightly decreased (L858R cells, Figure 3(e)) (E746-A750 del cells, Figure 3(f)) CMR over time. In contrast, wild-type EGFR-transfected H1299 cells showed consistent decrease of CMR with time (Figure 3(g)). At 120 min, the washout studies showed the higher retention of morpholino-[^131^I]IPQA in L858R (8.9 ± 0.2 CMR, Figure 3(e) red dot line) and E746-A750 del cells (12.6 ± 3.2 CMR, Figure 3(f) red dot line) than that in EGFR-transfected H1299 (5.7 ± 0.4 CMR, Figure 3(g) red dot line) and vector-transfected H1299 (6.6 ± 0.3 CMR, data not shown). The results of *in vitro* radiotracer uptake and washout studies of morpholino-[^131^I]IPQA are summarized in Table 1.

### 3.4. *In Vivo* PET Imaging of Morpholino-[^124^I]IPQA Exhibits Differentials of Accumulation in NSCLC Tumors with Different EGFR Expressions

**Figure 4:**
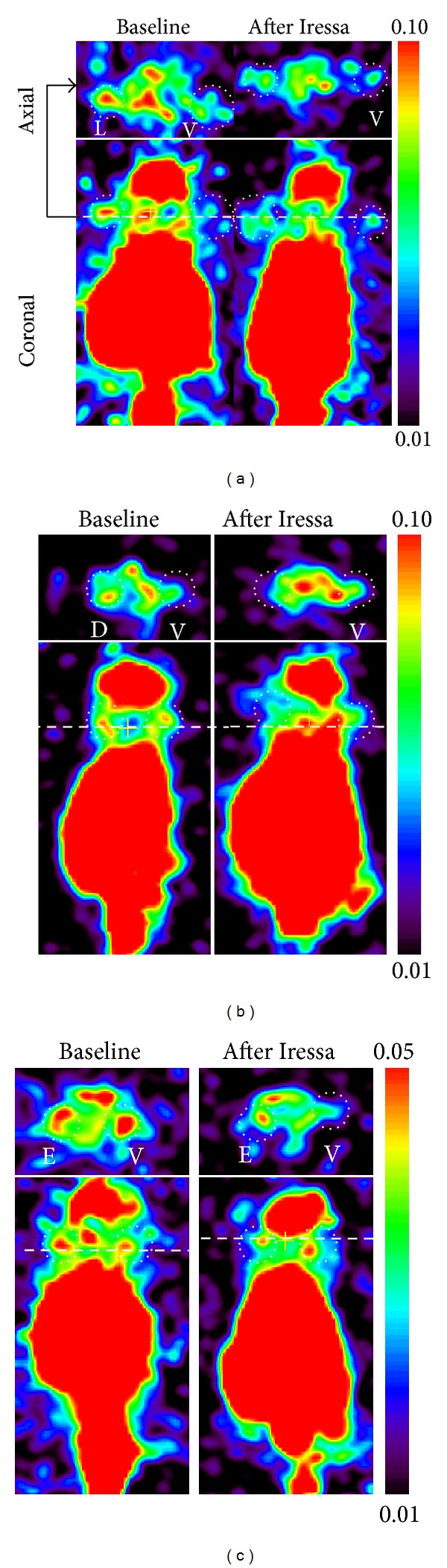
Reprehensive coronal and axial PET images obtained 24 hours after morpholino-[^124^I]IPQA administration in mice bearing L858R EGFR (a), E746-A750 del (b), wild-type EGFR-transfected (c), and EGFR-vector ((a)–(c)) subcutaneous tumor xenografts (dot circle). Color coding in the image is set to maximize the visualization of tumor in each projection. The different scale was used in (c). L: L858R EGFR; V: vector; D: E746-A750 del; E: EGFR-vector.

**Figure 5:**
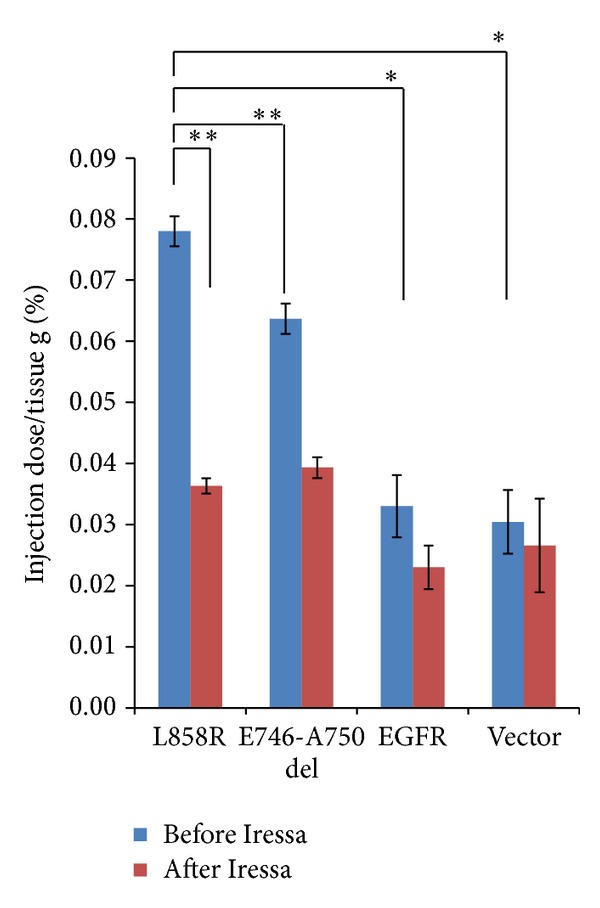
Quantification of accumulation of [^124^I]IPQA in different tumor xenografts at baseline (blue) and pretreated with Iressa (red), respectively. Error bars represent standard deviation; statically significant difference is indicated by an asterisk (*P < 0.05) or double asterisks (**P < 0.01).

*In vivo* PET imaging was performed in 18 mice (6 per tumor pair) before and after treatment with Iressa. MicroPET images demonstrated that highest accumulation level of morpholino-[^124^I]IPQA was observed in L858R tumor xenograft at 24 hours after radiotracer administration. The accumulation was 1.23-, 2.36-, and 3.08-folds higher than that of E746-A750 del, wild-type EGFR-transfected, and vector-transfected tumor xenograft in baseline group, respectively (Figures 4(a)–4(c), left panel, Figure 5). Pretreatment with Iressa (100 mg/kg 1 h before administration of morpholino-[^124^I]IPQA) results in 53% and 38% decrease in the accumulation of radiotracer in L858R and E746-A750 del tumors, respectively (Figures 4(a)-4(b) right panels, Figure 5). There was a similar accumulation of radiotracer in wild-type EGFR-transfected and vector-transfected tumor xenografts before and after Iressa treatment (Figures 4(a)–4(c); Figure 5). The results of tumor-to-vector ratio at 24 hours after administration of morpholino-[^124^I]IPQA was summarized in Table 5.

### 3.5. Biodistribution of Morpholino-[^131^I]IPQA Derivative in NSCLC Tumor-Bearing Mice

In the first hour, high % ID/g was observed in pancreas, kidney, stomach, lung, liver, and small and large intestines (Table 2). Four tumors expressing different levels of EGFR activity/expression had the peak radioactivity concentration in the first hour and then showed a gradual decrease over time; however, blood, heart, liver, and spleen showed a redistribution 24 hours after administration of morpholino-[^131^I]IPQA. The levels of radioactivity in blood or heart rapidly dropped to 0.1~0.2% ID/g one hour after radiotracer injection and progressively decreased during 4 to 48 hours. We also observed a rapid excretion of radiotracer by liver. This pattern of hepatobiliary clearance was followed by a fast increased radioactivity in blood and liver and subsequent clearance by kidneys.

All four tumor xenografts had similar accumulations of morpholino-[^131^I]IPQA at the first hour after injection of radiotracer. The L858R tumor xenograft had a longer retention time of morpholino-[^131^I]IPQA when compared to other 3 xenografts.

Up to 24 hours after injection of radiotracer, the tumor-to-blood (TBR) and tumor-to-muscle concentration ratios (TMR) were not distinguishable among four tumor xenografts (Tables 3 and 4; all other three cell lines were compared to L858R tumor cells at the same time point). The TBR and TMR in L858R tumor xenograft 24 hours after injection of radiotracer were 2.64 and 13.89, respectively (Tables 3 and 4). The E746-A750 del tumor xenograft had similar TBR and TMR compared to those of L858R group. The L858R tumor xenograft also had a better tumor-to-vector ratio 24 hours after administration of radiotracer when compared to those of other 2 xenografts (Table 5).

## 4. Discussion

Predicting the expression of EGFR mutation through noninvasive PET imaging with a specific EGFR kinase radiotracer would provide an assessment for the NSCLC patients who may be benefit from EGFR inhibitors therapeutic regiment. Previously, our collaborator reported that the PET imaging with morpholino-[^124^I]IPQA, which could irreversibly and specifically bind to active form of EGFR kinase, allowed for identification of tumors with high EGFR kinase signaling activity (i.e., A431 highly expressing EGFR in NSCLC and U87 del EGFR cells expressing EGFRvIII mutants in brain gliomas) [28].

In the current study, by using morpholino-[^124^I]IPQA with PET, we demonstrated that L858R and E746-A750 del EGFR mutated cells, which are the most frequent mutation in NSCLC [9], showed significant increased accumulation of radiotracer when compared to the wild-type EGFR-transfected and vector-transfected cells *in vitro* and *in vivo*. The locations of the L858R missense mutations are shown within the activating loop of the tyrosine kinase, whereas the in-frame deletion, E746-A750 del, is present within another loop which flanks the ATP cleft [14]. Those mutations are predicted to alter the position of these amino acids relative to that of phosphorylation status of the cells and the sensitivity of inhibitor (i.e., gefitinib).

The expression levels of phosphorylated EGFR in L858R and E746-A750 del EGFR mutated cells were similar, whereas wild-type EGFR-transfected cells showed the highest expression of total EGFR. Nevertheless, the mutation in active site of tyrosine kinase domain results in the enhanced EGFR signaling (phosphorylation status), which explains the increased accumulation of morpholino-[^131^I]IPQA in L858R and E746-A750 del EGFR mutated cells. Similar interpretation can also be applied to the preferential accumulation and retention of morpholino-[^124^I]IPQA in these mutant EGFR cells in *in vivo* PET imaging. 

In comparison with previous reports of EGFR imaging agents [22, 25, 26, 34, 35], the results of our *in vivo* PET imaging studies with morpholino-[^124^I]IPQA in mice bearing four tumor xenografts expressing different levels of phosphorylated EGFR are more selective. Predominant accumulations of morpholino-[^124^I]IPQA in EGFR-expressing L858R and E746-A750 del carcinoma tumor xenografts reflect the high level of phosphorylated EGFR expression and activity in those tumor cells, which are known to be responsive to therapy with small molecular inhibitors of EGFR (e.g., Gefitinib) [14]. The H1299 EGFR tumor xenograft expressing EGFR represented a model of therapy-resistant tumors. H1299 EGFR tumor had lower morpholino-[^124^I]IPQA accumulation and produced similar images before and after treatment with EGFR inhibitor, Gefitinib (Iressa).

Our results also provided more information of tissue distributions at later time points (24 and 48 hours after radiotracer administration). Selective accumulations of radiotracer in the L858R and E746-A750 del EGFR mutants were observed when compared to the tumors with wild-type EGFR or vector-transfected cells. Similar to the results from dynamic PET imaging obtained from 0 to 70 minutes after administration of morpholino-[^124^I]IPQA [28], the biodistribution data obtained from 1, 4, 24, and 48 hours after administration of morpholino-[^131^I]IPQA showed a rapid clearance of radiotracer from blood and other major tissues such as heart, brain, liver, intestines, and kidney. The results demonstrated that the nonspecific binding of the radiotracer was washed out rapidly and caused increased tumor-to-background ratio due to the irreversible and selective bindings to active state of EGFR kinase.

We also observed an unexpected high accumulation (3.2% ID/g at 24-hour time point) in pancreas at all time points. Mohammed et al. reported that gefitinib blocks EGFR signaling pathway in progression of pancreatic intraepithelial neoplasms (PanINs) to PDAC in conditional LSL-Kras^G12D/+^ transgenic mice model [36]. Kelley and Ko reported a significant survival benefit with the addition of the EGFR tyrosine kinase inhibitor erlotinib to gemcitabine chemotherapy for the first-line treatment of patients with advanced pancreatic cancer [37]. These results demonstrated that morpholino-[^131^I]IPQA was entrapped in normal pancreatic cell (i.e., ductal cells and the islets of Langerhans) [38].

However, despite the lower lipophilicity of morpholino-[^124^I]IPQA compared to previously reported EGFR imaging compounds such as the N-{4-[(4,5-dichloro-2-fluorophenyl)amino]quinazolin-6-yl}-acrylamide, 4-[(3,4-dichloro-6-fluorophenyl)amino]-6,7-dimethoxyquinazoline, and 4-(3-bromoanilino)-6,7-dimethoxyquinazoline [22, 25, 26, 34, 35], the morpholino-[^124^I]IPQA still exhibited a significant hepatobiliary clearance. Also, high accumulation of the radiotracer in abdomen area over time would decrease the possibility of using this radiotracer for imaging primary site of NSCLC in animal model or for colorectal metastases. The further improvement could focus on the optimization of its pharmacokinetic properties by additional chemical derivatization (i.e., decrease lipophilicity and extraction by the liver, increase the half-life in plasma and accumulation amount/retention time in tumor tissue, and increase water solubility and renal clearance for a better background-to-target ratio).

The relatively low resolution of microPET system (spatial resolution ~1.8 mm) may not allow for assessing the heterogeneity of morpholino-[^124^I]IPQA radioactivity accumulation. Therefore, micro-PET/CT and autoradiography (with 10–30 *μ*M in plane resolution) are suitable to study the heterogeneity of morpholino-[^124^I]IPQA-derived radioactivity accumulation and to compare it with the immunohistochemically stained (adjacent) tissue sections for the total EGFR or phosphorylated EGFR (Tyrosine 1068).

As a breakthrough of NSCLC treatment management in 2004, Lynch et al. [14] first reported that specific mutations in *EGFR *gene in NSCLC patients were correlated with clinical responsiveness to the tyrosine kinase inhibitor gefitinib. These findings were confirmed by Pao et al. in the tumor cells from the patients with L858R mutation but not for L747-S752 del EGFR mutant [14]. The study results from Sordella et al. also supported that EGF-independent autophosphorylation in these mutations caused high sensitivity to gefitinib and selectively activated downstream pathways [39]. Tracy et al. also proved that gefitinib-induced apoptosis in the L858R mutant lead dramatic response to gefitinib [40]. The authors also concluded that the affinity for the L858R mutant to gefitinib was 20-fold higher than that for wild type, which could be explained by its tighter binding to active conformation of the tyrosine kinase domain. Also, it could be expected to predict the efficacy in the treatment of the subgroup with gefitinib. Tracy et al. suggested that targeted agents developed to inhibit AKT pathway may be therapeutically more effective than those designed to inhibit the ERK 1/2 pathway in patients whose tumors contain EGFR L858R if the AKT pathway is consistently active [40]. Moreover, Brognard et al. reported that constitutive AKT activation has also been associated with resistance to chemotherapy and radiation in NSCLC cell lines [41]. Further study could be the mono/combinations of chemotherapy with gefitinib or inhibitors of the AKT pathway which should be tested *in vitro *in cell lines and *in vivo *in tumor xenografts with EGFR L858R mutations to determine whether these may be additive or synergistic.

In conclusion, an enhanced binding of morpholino-[^124/131^I]IPQA derivatives to the ATP binding site of mutant kinase of L858R or E746-A750 del EGFR mutant warrants that PET imaging with morpholino-[^124^I]IPQA has a potential for identification of tumors with high EGFR kinase activity in NSCLC and for the monitoring or selection of individual therapies with EGFR inhibitors. Further optimization of this class of radio compound would be necessary in order to lower lipophilicity and reduce hepatobiliary clearance.

## Figures and Tables

**Table 1:** Pharmacokinetics of morpholino-[^131^I]IPQA accumulation and washout in different tumor cells *in vitro*.

Tumor cells	L858R	E746-A750 del	EGFR	Vector
Plateau level at 60 minutes^1^	3.12 ± 0.304	1.57 ± 0.17	1.47 ± 0.001	1.47 ± 0.001
Washout at 60 minutes^2^	1.78 ± 0.147*	1.10 ± 0.16	1.17 ± 0.001	1.17 ± 0.001
Washout at 21 minutes	1.09 ± 0.124*	1.16 ± 0.20	0.55 ± 0.000*	0.55 ± 0.000*

^1^Volume of distribution at equilibrium expressed as cell-to-medium accumulation ratio (average ± standard error). ^2^Coefficient *B* (slope) of the exponential washout = *A* × exp^(−BX)^.

**Table 2:** Biodistribution of accumulated radioactivity (%ID/g) in different tissues at the designed time points after administration of morpholino-[^131^I]IPQA.

Tissue	Radioactivity (%injection dose/tissue g)			
	1 hr	4 hr	24 hr	4 hr
Blood	0.124 ± 0.000	0.076 ± 0.000	0.030 ± 0.000	0.009 ± 0.000
Lung	1.319 ± 0.001	0.776 ± 0.001	0.239 ± 0.000	0.119 ± 0.000
Heart	0.240 ± 0.002	0.191 ± 0.001	0.076 ± 0.000	0.676 ± 0.001
Stomach	1.330 ± 0.001	1.302 ± 0.002	1.407 ± 0.012	0.299 ± 0.000
Liver	1.241 ± 0.002	0.500 ± 0.000	0.424 ± 0.000	1.063 ± 0.001
Spleen	0.560 ± 0.009	0.303 ± 0.000	0.225 ± 0.001	0.486 ± 0.001
Pancreas	5.443 ± 0.002	5.334 ± 0.002	3.232 ± 0.004	0.389 ± 0.002
Small intestine	0.858 ± 0.001	0.774 ± 0.004	0.135 ± 0.000	0.044 ± 0.000
Large intestine	0.807 ± 0.002	0.289 ± 0.000	0.301 ± 0.000	0.021 ± 0.000
Kidney	4.785 ± 0.001	3.772 ± 0.008	1.431 ± 0.001	0.523 ± 0.000
Bone	0.190 ± 0.002	0.109 ± 0.000	0.062 ± 0.000	0.04 ± 0.000
Muscle	0.034 ± 0.000	0.025 ± 0.000	0.009 ± 0.000	0.009 ± 0.000
Brain	0.010 ± 0.000	0.008 ± 0.000	0.003 ± 0.000	0.002 ± 0.000
L858R	0.280 ± 0.000	0.251 ± 0.001	0.125 ± 0.000	0.037 ± 0.000
E746-A750 del	0.304 ± 0.000	0.195 ± 0.000	0.100 ± 0.000	0.052 ± 0.000
EGFR	0.347 ± 0.000	0.208 ± 0.000	0.047 ± 0.000	0.036 ± 0.000
Vector	0.222 ± 0.000	0.143 ± 0.000	0.085 ± 0.001	0.123 ± 0.002

Data shown as average ± standard deviation (*n* = 3 per tumor pair).

**Table 3:** Tumor-to-blood ratio in different tumor cells in *in vitro* study.

	1 hr	4 hr	24 hr	48 hr
L858R/blood	2.25 ± 0.001	3.75 ± 0.001	2.64 ± 0.000	3.98 ± 0.000*
E746-A750 del/blood	2.45 ± 0.000	2.96 ± 0.001	2.68 ± 0.000	5.55 ± 0.002*
Wild-type EGFR/blood	2.79 ± 0.001*	2.91 ± 0.000	1.22 ± 0.000	3.87 ± 0.001*
Vector/blood	1.79 ± 0.001	1.97 ± 0.000	2.37 ± 0.001	13.12 ± 0.000

Data shown as average ± standard deviation (*n* = 3 for each tumor pair). All other three cell lines were compared to L858R tumor cells at the same time point, and statistically significant difference (*P < 0.05) is indicated by an asterisk.

**Table 4:** Tumor-to-muscle ratio in different tumor cells in *in vitro* study.

	1 hr	4 hr	24 hr	48 hr
L858R/muscle	8.24 ± 0.001	10.04 ± 0.001*	13.99 ± 0.001*	4.11 ± 0.000*
E746-A750 del/muscle	8.94 ± 0.001*	7.80 ± 0.001	11.11 ± 0.001*	5.78 ± 0.001*
Wild-type EGFR/muscle	10.21 ± 0.00*	8.32 ± 0.002*	5.22 ± 0.002*	4.00 ± 0.001*
Vector/muscle	6.53 ± 0.001	5.36 ± 0.000	9.44 ± 0.001	13.67 ± 0.001*

Data shown as average ± standard deviation (*n* = 3 for each tumor pair). All other three cell lines were compared to L858R tumor cells at the same time point, and statistically significant difference (*P < 0.05) is indicated by an asterisk.

**Table 5:** Tumor-to-vector ratio in different tumor cells in *in vitro* study of morpholino-[^131^I]IPQA and *in vivo* study of morpholino-[^124^I]IPQA.

	PET		Biodistribution
	Baseline	Iressa	Baseline
L858R/vector	3.12 ± 0.304	1.57 ± 0.17	1.47 ± 0.001
E746-A750 del/vector	1.78 ± 0.147*	1.10 ± 0.16	1.17 ± 0.001
EGFR/vector	1.09 ± 0.124*	1.16 ± 0.20	0.55 ± 0.000*

Data shown as average ± standard deviation (*n* = 3 for each tumor pair). All other three cell lines were compared to L858R tumor, and statistically significant difference (*P < 0.05) is indicated by an asterisk.

## References

[1] ACS http://www.cancer.org/cancer/lungcancer-non-smallcell/detailedguide/non-small-cell-lung-cancer-key-statistics.

[2] Burris HA (2009). Shortcomings of current therapies for non-small-cell lung cancer: unmet medical needs. *Oncogene*.

[3] Yarden Y (2001). The EGFR family and its ligands in human cancer: signalling mechanisms and therapeutic opportunities. *European Journal of Cancer*.

[4] Gazdar AF (2009). Activating and resistance mutations of EGFR in non-small-cell lung cancer: role in clinical response to EGFR tyrosine kinase inhibitors. *Oncogene*.

[5] Sharma SV, Bell DW, Settleman J, Haber DA (2007). Epidermal growth factor receptor mutations in lung cancer. *Nature Reviews Cancer*.

[6] Reade CA, Ganti AK (2009). EGFR targeted therapy in non-small cell lung cancer: potential role of cetuximab. *Biologics*.

[7] Fukuoka F (2003). Multi-institutional randomized phase II trial of gefitinib for previously treated patients with advanced non-small-cell lung cancer (The IDEAL 1 Trial). *Journal of Clinical Oncology*.

[8] Kris MG, Natale RB, Herbst RS (2003). Efficacy of gefitinib, an inhibitor of the epidermal growth factor receptor tyrosine kinase, in symptomatic patients with non-small cell lung cancer: a randomized trial. *Journal of the American Medical Association*.

[9] Paez JG, Jänne PA, Lee JC (2004). EGFR mutations in lung, cancer: correlation with clinical response to gefitinib therapy. *Science*.

[10] Huang S-F, Liu H-P, Li L-H (2004). High frequency of epidermal growth factor receptor mutations with complex patterns in non-small cell lung cancers related to gefitinib responsiveness in Taiwan. *Clinical Cancer Research*.

[11] Thatcher N, Chang A, Parikh P (2005). Gefitinib plus best supportive care in previously treated patients with refractory advanced non-small-cell lung cancer: results from a randomised, placebo-controlled, multicentre study (Iressa Survival Evaluation in Lung Cancer). *The Lancet*.

[12] Shepherd FA, Tsao M-S (2006). Unraveling the mystery of prognostic and predictive factors in epidermal growth factor receptor therapy. *Journal of Clinical Oncology*.

[13] Riely GJ, Pao W, Pham D (2006). Clinical course of patients with non-small cell lung cancer and epidermal growth factor receptor exon 19 and exon 21 mutations treated with gefitinib or erlotinib. *Clinical Cancer Research*.

[14] Lynch TJ, Bell DW, Sordella R (2004). Activating mutations in the epidermal growth factor receptor underlying responsiveness of non-small-cell lung cancer to gefitinib. *The New England Journal of Medicine*.

[15] Pao W, Miller VA, Politi KA (2005). Acquired resistance of lung adenocarcinomas to gefitinib or erlotinib is associated with a second mutation in the EGFR kinase domain. *PLoS Medicine*.

[16] Jackman DM, Yeap BY, Sequist LV (2006). Exon 19 deletion mutations of epidermal growth factor receptor are associated with prolonged survival in non-small cell lung cancer patients treated with gefitinib or erlotinib. *Clinical Cancer Research*.

[17] Riely GJ, Politi KA, Miller VA, Pao W (2006). Update on epidermal growth factor receptor mutations in non-small cell lung cancer. *Clinical Cancer Research*.

[18] Giaccone G (2005). Epidermal growth factor receptor inhibitors in the treatment of non-small-cell lung cancer. *Journal of Clinical Oncology*.

[19] Jänne PA (2005). Ongoing first-line studies of epidermal growth factor receptor tyrosine kinase inhibitors in select patient populations. *Seminars in Oncology*.

[20] Gelovani JG (2008). Molecular imaging of epidermal growth factor receptor expression-activity at the kinase level in tumors with positron emission tomography. *Cancer and Metastasis Reviews*.

[21] Dissoki S, Aviv Y, Laky D, Abourbeh G, Levitzki A, Mishani E (2007). The effect of the [^18^F]-PEG group on tracer qualification of [4-(phenylamino)-quinazoline-6-YL]-amide moiety-An EGFR putative irreversible inhibitor. *Applied Radiation and Isotopes*.

[22] Mishani E, Abourbeh G, Rozen Y (2004). Novel carbon-11 labeled 4-dimethylamino-but-2-enoic acid [4-(phenylamino)-quinazoline-6-yl]-amides: potential PET bioprobes for molecular imaging of EGFR-positive tumors. *Nuclear Medicine and Biology*.

[23] Mishani E, Abourbeh G (2007). Cancer molecular imaging: radionuclide-based biomarkers of the epidermal growth factor receptor (EGFR). *Current Topics in Medicinal Chemistry*.

[24] Mishani E, Hagooly A (2009). Strategies for molecular imaging of epidermal growth factor receptor tyrosine kinase in cancer. *Journal of Nuclear Medicine*.

[25] Bonasera TA, Ortu G, Rozen Y (2001). Potential^18^F-labeled biomarkers for epidermal growth factor receptor tyrosine kinase. *Nuclear Medicine and Biology*.

[26] Ortu G, Ben-David I, Rozen Y (2002). Labeled EGFr-TK irreversible inhibitor (ML03): *in vitro* and *in vivo* properties, potential as pet biomarker for cancer and feasibility as anticancer drug. *International Journal of Cancer*.

[27] Abourbeh G, Dissoki S, Jacobson O (2007). Evaluation of radiolabeled ML04, a putative irreversible inhibitor of epidermal growth factor receptor, as a bioprobe for PET imaging of EGFR-overexpressing tumors. *Nuclear Medicine and Biology*.

[28] Pal A, Glekas A, Doubrovin M (2006). Molecular imaging of EGFR kinase activity in tumors with ^124^I-labeled small molecular tracer and positron emission tomography. *Molecular Imaging and Biology*.

[29] Su H, Seimbille Y, Ferl GZ (2008). Evaluation of [^18^F]gefitinib as a molecular imaging probe for the assessment of the epidermal growth factor receptor status in malignant tumors. *European Journal of Nuclear Medicine and Molecular Imaging*.

[30] Wang H, Yu J-M, Yang G-R (2007). Further characterization of the epidermal growth factor receptor ligand 11C-PD153035. *Chinese Medical Journal*.

[31] Memon AA, Jakobsen S, Dagnaes-Hansen F, Sorensen BS, Keiding S, Nexo E (2009). Positron emission tomography (PET) imaging with [^11^C]-labeled erlotinib: a micro-PET study on mice with lung tumor xenografts. *Cancer Research*.

[32] Liu N, Li M, Li X (2009). PET-based biodistribution and radiation dosimetry of epidermal growth factor receptor-selective tracer ^11^C-PD153035 in humans. *Journal of Nuclear Medicine*.

[33] Deng W-P, Yang WK, Lai W-F (2004). Non-invasive *in vivo* imaging with radiolabelled FIAU for monitoring cancer gene therapy using herpes simplex virus type 1 thymidine kinase and ganciclovir. *European Journal of Nuclear Medicine and Molecular Imaging*.

[34] Fredriksson A, Johnström P, Thorell J-O (1999). *In vivo* evaluation of the biodistribution of 11C-labeled PD153035 in rats without and with neuroblastoma implants. *Life Sciences*.

[35] Shaul M, Abourbeh G, Jacobson O (2004). Novel iodine-124 labeled EGFR inhibitors as potential PET agents for molecular imaging in cancer. *Bioorganic and Medicinal Chemistry*.

[36] Mohammed A, Janakiram NB, Li Q (2010). The epidermal growth factor receptor inhibitor gefitinib prevents the progression of pancreatic lesions to carcinoma in a conditional LSL-Kras^G12D/+^ transgenic mouse model. *Cancer Prevention Research*.

[37] Kelley RK, Ko AH (2008). Erlotinib in the treatment of advanced pancreatic cancer. *Biologics*.

[38] Ueda S, Ogata S, Tsuda H (2004). The correlation between cytoplasmic overexpression of epidermal growth factor receptor and tumor aggressiveness: poor prognosis in patients with pancreatic ductal adenocarcinoma. *Pancreas*.

[39] Sordella R, Bell DW, Haber DA, Settleman J (2004). Gefitinib-sensitizing EGFR mutations in lung cancer activate anti-apoptotic pathways. *Science*.

[40] Tracy S, Mukohara T, Hansen M, Meyerson M, Johnson BE, Jänne PA (2004). Gefitinib induces apoptosis in the EGFRL858R non-small-cell lung cancer cell line H3255. *Cancer Research*.

[41] Brognard J, Clark AS, Ni Y, Dennis PA (2001). Akt/pbotein kinace B is constitutively active in non-small cell lung cancer cells and promotes cellular survival and resistance to chemotherapy and radiation. *Cancer Research*.

